# Efficiency Analysis of Fuel Cell Components with Ionic Poly-Arylether Composite Membrane

**DOI:** 10.3390/membranes12121238

**Published:** 2022-12-07

**Authors:** Hsin-Yi Wen, Guang-Hsiang Wang, Mei-Ying Chang, Wen-Yao Huang, Tung-Li Hsieh

**Affiliations:** 1 Department of Chemical and Materials Engineering, National Kaohsiung University of Science and Technology, Kaohsiung 80778, Taiwan; 2 Department of Photonics, National Sun Yat-sen University, Kaohsiung 80424, Taiwan; 3 Department of Electronics Engineering, National Kaohsiung University of Science and Technology, Kaohsiung 80778, Taiwan

**Keywords:** polyethylene glycol, hydrogen bonding, dimensional stability, thermal stability, physical cross-linking

## Abstract

We use polyethylene glycol as an additive to explore how the hydrogen bonding of this additive changes the properties of SA8 blended sulfonated polyetheretherketone (SPEEK) composite films. We mixed a 5%wt polyethylene glycol solution into a 12.5%wt SA8 solution, and then prepared a film with a total weight of 40g at a ratio of 1:99. The SA8 (PEG) solution was prepared and then mixed with 5%wt SPEEK solution, and a film-forming solution with a total weight of 8g in different mixing ratios was created. Polyethylene glycol (PEG) was mixed into the sulfonated polyarylether polymer SA8 to form physical cross-linking. Therefore, the sulfonated polyether ether ketone SPEEK was mixed in, and it exhibited good thermal stability and dimensional stability. However, there was some decrease in proton conductivity as the proportion of SPEEK increased. Although SPEEK mixed with sulfonated polymer reduces the proton conductivity, the physical cross-linking of PEG can improve the proton conductivity of the composite membrane, and adding SPEEK can not only solve the problem of the high sulfonation film swelling phenomenon, it can also improve the dimensional stability of the film through the hydrogen bonding force of PEG and obtain a composite film with excellent properties.

## 1. Introduction

The aim of composite polymer membranes is to determine how to produce a composite membrane with stable miscibility and good electrical conductivity. The sulfonated aromatic ring-based composite membrane is one of the hot spots due to Tae-Hyun Kim’s research team at Incheon University in South Korea, Santoshkumar D Bhat’s research team from the Central Electro Chemical Research Institute in India and Sanjay Bisht and Sasikumar Balaguru’s research team in India. In 2015, Tae-Hyun Kim’s research team at Incheon University in South Korea developed a new type of cross-linked polymer blend (CPB) film [1]. The ionic polymer composite membrane blend was achieved based on sulfonated poly(ether sulfone) (sPES) and poly(p-benzimidazole) (p-PBI). In 2016, Santoshkumar D. Bhat’s research team from the Central Electro Chemical Research Institute in India developed a sulfonated poly(bis(phenoxy) phosphazene (sPOP) blend membrane [2]. In sPOP-sulfonated poly(ether ketone)) (sPOP-sPEEK) blend membranes, the tensile strength increases with the increase in sPOP content in the film. This phenomenon might be due to the strong hydrogen bonds between the sulfonate in the blend membrane and the nitrogen in the phosphine structure, which form physical cross-links. In 2020, the Indian research team headed by Sanjay Bisht and Sasikumar Balaguru reported the development of several blended membranes: sPEEK blended with SiO_2_ and sulfonated SiO_2_ (S-SiO_2_) blended with metal–organic framework (MOF-5) [3]. The results demonstrated the water absorption, ion-exchange capacity (IEC), and dimensional stability of various composite membranes. From the data, sPEEK was observed to have very good dimensional stability, which was improved with the increase in SiO_2_ with water absorption capacity. Therefore, the hydrogen bonding of the sPEEK and S-SiO_2_ blend effectively controlled the water absorption capacity of the film and further improved its dimensional stability. In terms of thermal stability, sPEEK exhibited good thermal stability with a simple cracking mechanism. However, with the mixing of other materials, the derivatives generated by the interaction between these materials at high temperature increases the complexity of the cracking mechanism of the film. This also confirms that the force generated by the blending of the materials directly affects the properties of the film. In 2004, the Canadian research team headed by Serge Kaliaguine reported the preparation of composite polymer membranes by the addition of ethylene glycol to sPEEK [4]. Through the formation of cross-links between the sulfonic acid groups on sPEEK and ethylene glycol, a grafting network was effectively established. This study showed that the sPEEK composite membrane became more flexible owing to the “soft” chain characteristics of ethylene glycol, which effectively improved the mechanical properties of the film. However, with the increase in ethylene glycol content, excessive crosslinking was formed, which rendered the membrane more fragile; hence, controlling the amount of ethylene glycol used is crucial. Therefore, in this study, polyethylene glycol (PEG) was used as an additive to optimize the proton transport property of the membrane. Moreover, the self-made SA8 structural polymer was introduced [5], and the effect of the hydrogen bonding force of PEG on the performance of SA8 was investigated. In terms of proton conductivity, the proton conductivity of SA8 under high and low humidity conditions was investigated due to the physical cross-linking of PEG. The hydrogen bonding force formed by PEG, with “soft” chain properties, and SA8 was used to evaluate the mechanical properties and dimensional stability of SA8. A series of hybrid inorganic–organic ion exchange membranes (IEMs) exhibit the physical cross-linking that was demonstrated in the literature: a higher coulombic efficiency, a higher energy efficiency, a higher capacity retention, the better mechanical properties and dimensional stability [6,7].

After obtaining PEG added with SA8, sPEEK was blended to study the effect of the hydrogen bonding force of PEG on the performance of the SA8-sPEEK composite membrane, as well as to compare the performance of the SA8-sPEEK composite membrane without PEG and explore the change of the membrane proton conductivity through the physical crosslinking of PEG between the two macromolecules. sPEEK has high dimensional stability, which improves the performance of SA8.

## 2. Materials and Methods

### 2.1. Preparation of SA8 Solution

**Figure 1:**
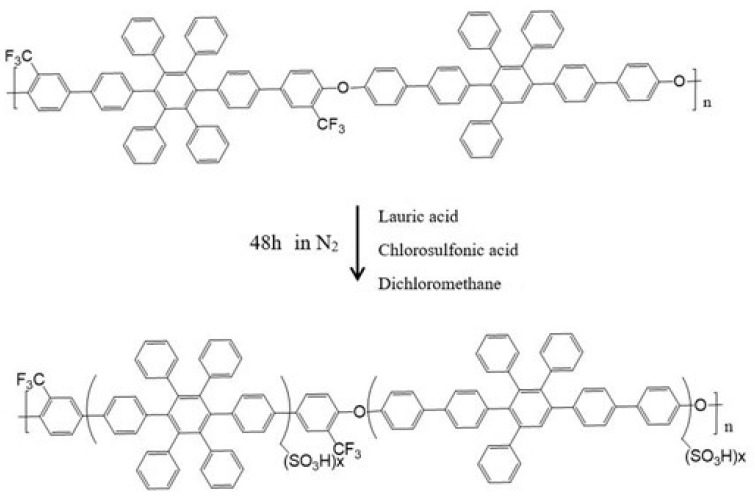
Diagram of SA8 polymer sulfonation.

First, The sulfonation fluorinated poly(aryl ether)s (SA8) with the linkage of ether (–O–) between fluorinated monomers and bisphenols that was reported by Chun-Che Lee, of which the sulfonation degree of SA8 is 81.20% [8], is a series of materials that is known to be rather thermally stable, whereby the thermal decomposition temperatures of polymers are as high as 560 °C. In 2015, our laboratory developed the SA8 series of alternating polyphenylene ring polyaromatic ether polymers. The IEC of SA8 was above 2, the water absorption capacity was above 30% at a temperature of 90 °C, the dimensional stability exceeded 30%, and the proton conductivity reached 255 mS/cm, which indicated that SA8 has high water absorption capacity and proton conductivity. However, SA8 is limited in component provisioning because of its poor mechanical properties. We placed the SA8 solid in an oven for 24 h to dry; subsequently, we placed a magnet in a beaker, added 25 g of the dried SA8 solid and 300 g of methanol, and stirred for several hours until it was completely dissolved. The solution was filtered with cotton filter to partially remove cross-linked polymers and impurities. After repeated filtration, the SA8 solution was weighed and concentrated under reduced pressure to form a high-viscosity solution with a weight of approximately 100 g. The percentage weight concentration of the concentrated SA8 solution was approximately 25 wt%. A total of 40 g of concentrated SA8 solution and 40 g of dimethyl sulfoxide (DMSO) [9] solvent were used to prepare the SA8 film-forming solution (FFS) [10] of 12.5 wt%, whereby the weighted ratio of the mixed solvent was approximately MeOH:DMSO = 1:4. The sulfonated SA8 polymer structure is shown in Figure 1.

### 2.2. Preparation of Polyethylene Glycol Solution

**Figure 2:**
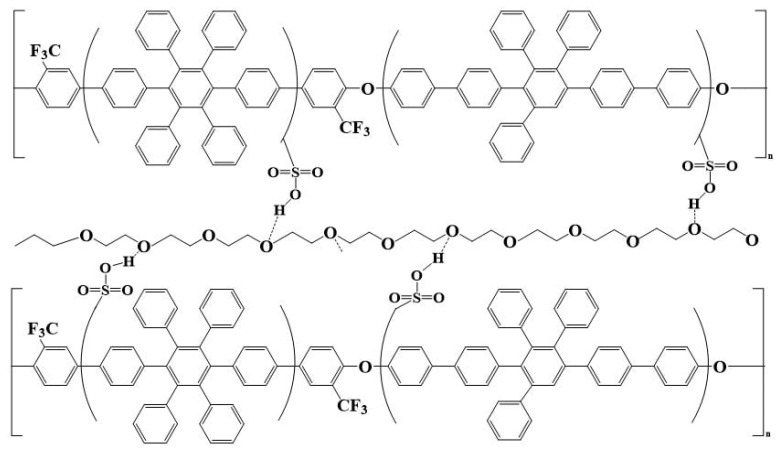
Schematic of physical crosslinking of sulfonated polymers.

The PEG powder was dried in an oven for 24 h. Moreover, 5 g of dried PEG powder and 95 g of methanol as the solvent were inserted in a beaker with a magnet. The beaker was sealed with parafilm and a leak-proof tape and stirred continuously to prepare a PEG solution with a percentage weight concentration of 5%. At present, the proton exchange membranes contain hydrocarbon ionic polymers as the main structural component. The degree of sulfonation is usually increased to improve the proton conductivity. However, an excessively high degree of sulfonation deteriorates the dimensional stability of the membrane under high temperature and high humidity environments, which results in film swelling caused by excessive water absorption that is disadvantageous in fuel cells. As shown in Figure 2, the hydrogen bonding force of the PEG structure effectively restrains the sulfonate, and the polymer structure is physically cross-linked [11] by hydrogen bonds. Hence, appropriate addition of PEG improves the dimensional stability of the membrane; PEG has a “soft” chain structure and is often used as a plasticizer in engineering, which improves the elongation at the break of the membrane and increases the flexibility of the film in terms of its mechanical properties. In addition to the optimization of the dimensional stability of the sulfonated poly(aryl ether) polymer, another focus of this experiment is the change in proton transport through the physical crosslinking of the sulfonated poly(aryl ether) polymer via hydrogen bonding. We investigated whether the presence or amount of physical crosslinking affected the change in the proton transport properties of the membrane, according to the IEC of the membrane and proton conductivity under high and low humidity conditions.

### 2.3. Preparation of SA8 and PEG Film-Forming Solution

The 5 wt% PEG solution [12] was mixed with the 12.5 wt% SA8 solution. The total weight of the FFS was 8 g; this solution was sealed and stirred for 2 h at 80 °C, after which it was poured evenly on a large-area film-forming glass. An automatic film coater was utilized to coat and form a film at a fixed rate, and the finished film was placed in an oven at 80 °C to dry for 24 h. After drying, the film was removed and dissected into several sizes. Subsequently, the film was placed in a 1 M HCl aqueous solution for 24 h (replaced with fresh HCl aqueous solution every 12 h) and removed after soaking in acid. The film was washed with deionized (DI) water, which became neutral (tested with litmus paper); finally, the film was dried using filter paper and placed in a vacuum oven at 80 °C for moisture removal and drying. The solid contents of SA8 and PEG FFS are listed in Table 1.

### 2.4. Preparation of sPEEK Solution

**Figure 3:**
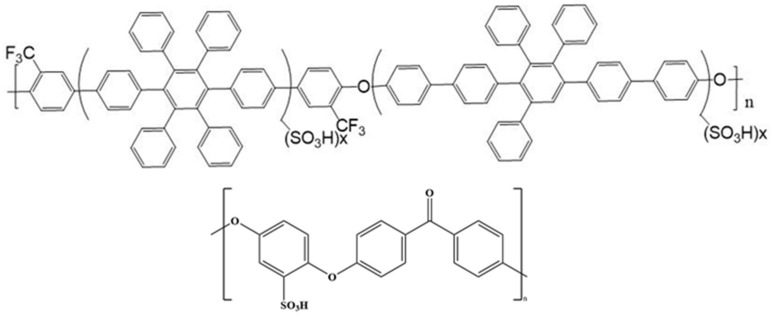
Structure of SA8 and sPEEK polymer composite membrane.

The sPEEK was placed in an oven for 24 h. Moreover, 5 g of dried sPEEK was placed in a beaker containing 95 g of DMSO solvent and a magnetic stirrer, sealed with parafilm and leak-stopping tape, heated to 80 °C and stirred for 24 h. After dispersing sPEEK in the solvent, suction filtration was used to filter out the impurities to prepare a 5 wt% sPEEK solution. In this experiment, sPEEK was introduced to mix with the sulfonated polyaromatic ether polymer SA8 developed in the laboratory, as shown in Figure 3. After the engineering material PEEK [13] was sulfonated, it exhibited high dimensional and thermal stabilities of the original engineering material and, also, good film-forming properties. The cost of PEEK thermoplastic is low, and the reaction conditions for sulfonation are relatively simple; hence, composite polymer film blended with sPEEK is discussed in numerous pieces of the literature. SPEEK with a high degree of sulfonation easily causes the polymer to fail to precipitate due to hydrolysis, whereas sPEEK with a low degree of sulfonation has poorer proton conductivity. Therefore, sPEEK was blended with the sulfonated polyaromatic ether polymer SA8 to obtain a polymer composite membrane with dual material properties.

### 2.5. Preparation of SA8 and sPEEK Film-Forming Solution

The 5 wt% sPEEK solution was mixed with the 12.5 wt% SA8 solution [14], and the total weight of the FFS was 8 g; this solution was sealed and stirred for 2 h at 80 °C. After heating and stirring the FFS, it was evenly poured on a large-area film-forming glass, and an automatic film coater was used to coat and form a film at a fixed rate; the finished film was placed into an oven at 80 °C for 24 h. After drying, the film was removed and dissected into several sizes. In sequence, it was placed in a 1 M HCl aqueous solution for 24 h (replaced with fresh HCl aqueous solution every 12 h), and the film was removed after soaking in acid. The film was brushed with DI water until the DI water became neutral (tested with litmus paper); finally, the film was pressed to dry with filter paper and placed in a vacuum oven at 80 °C for moisture removal and drying. The solid contents of SA8 and sPEEK FFS are listed in Table 2.

### 2.6. Preparation of SA8, PEG and sPEEK Film-Forming Solution

Initially, 5 wt% of PEG solution was mixed with 12.5 wt% of SA8 solution; the total weight of the FFS was 40 g, it was prepared in a ratio of 1:99 and was sealed and stirred for 2 h at 80 °C. The solution of SA8(PEG) was prepared, and the corresponding solid contents are listed in Table 3. Subsequently, the prepared SA8(PEG) solution was mixed with 5 wt% of sPEEK solution, and the total weight of the FFS was 8 g, which was prepared with different blending ratios and sealed and stirred at 80 °C for 2 h. The blending ratio of the FFS is presented in Table 4. After heating and stirring the FFS, it was evenly poured on a large-area film-forming glass, and an automatic film coater was used to coat and form a film at a fixed rate; the finished film was placed in an oven at 80 °C for 24 h. After drying, the film was removed and cut into several sizes. Next, the film was placed in a 1 M HCl aqueous solution for 24 h (replaced with fresh HCl aqueous solution every 12 h) and removed after soaking in acid. The film was cleaned with DI water until the DI water became neutral (tested with litmus paper); finally, the film was dried using filter paper and placed in a vacuum oven at 80 °C for moisture removal and drying [15].

### 2.7. Preparation of Fuel Cell Components

**Figure 4:**
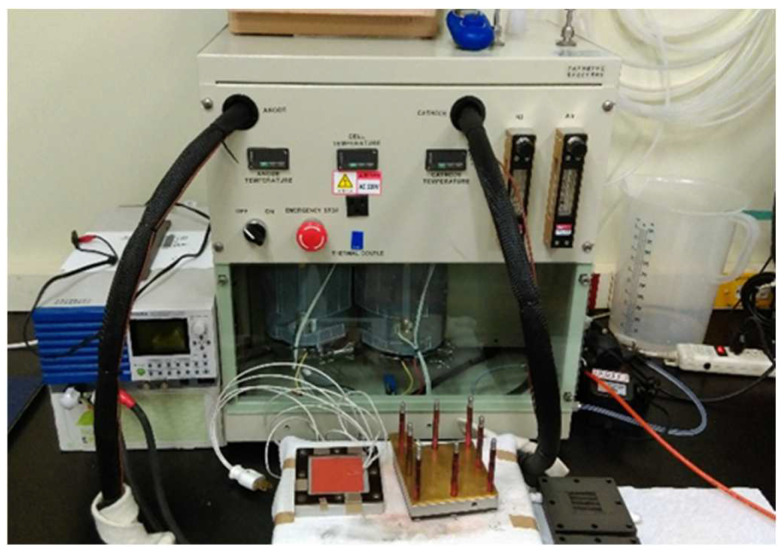
A machine for measuring fuel cells.

Figure 4 shows the fuel cell original machine in the laboratory. The model is TEI-P300NS 300W PEMFC, and the fuel cell component performance measurement machine is composed of a combination of multiple instruments, namely, hydrogen and oxygen fuel suppliers and gas flow channel valves, electronic load and performance measuring instrument, external temperature heater, heating belt or heating patch. The gas flow valve sets the required intake volume, (hydrogen: 0.2 L/min), (oxygen: 0.4 L/min), and then, the outside of the battery is covered by the heating belt and the heating patch, and the external temperature of the battery element is heated. After heating the internal temperature to 80 °C through the hydrogen-oxygen fuel supplier, it will be activated. The condition is to, first, set a constant voltage of 0.9 V to activate for 30 s and, then, fix a constant voltage of 0.2 V to activate for 60 s. The two steps are repeated 30 times as a cycle, and finally the approximate open circuit voltage value is 0.95 V, which is then decreased by 0.025 V every 10 s, and the current density and performance are scanned.

The previously mentioned sulfonated polymers of different material formulations were cut into 3 cm × 3 cm pieces for fuel cell component measurement. The preparation formula of the catalyst layer in this experiment was prepared using Nafion [16,17] D520 (5 wt%), methanol, DI water and Pt/C (40 wt%). After the catalyst formula was carefully prepared, the catalyst was placed in an ultrasonic oscillator for 1 h, and a magnetic stirrer was added to rotate and stir the slurry to ensure even dispersion of the catalyst. The catalyst slurry was subsequently poured into a feeding tube, the temperature of the spraying chamber was controlled at 80 °C, and the catalyst was uniformly sprayed on the proton exchange membrane. Subsequently, the Pt loading of the cathode and anode was converted by weighing and combined with the gas diffusion layer (GDL) to form a membrane electrode assembly (MEA). The MEA was assembled on the fuel cell [18,19] component, at which the operation temperature was controlled at 80 °C, the relative humidity was 100%, and the cathode and anode were fed with pure oxygen and pure hydrogen, respectively, for measurement. Pt and D520 were prepared according to the solids content weight ratio of 1:1 such that the experimental catalyst slurry contained Pt/C (0.218 g), Nafion D520 (1.757 g), methanol (23.015 g), and DI water (6.03 g). In terms of the parameters of the spraying component, the ratio of Pt loading of the cathode to the anode was 2:1. Therefore, the spraying procedures of the cathode and anode had 160 steps and 80 steps, respectively. The spraying area of the catalyst was 1 cm × 1 cm. The thickness of the GDL was 0.235 mm and the cut area was 1.4 cm × 1.4 cm. The ratio of hydrogen to oxygen intake in the fuel cell was 2:1.

### 2.8. Instrument Measurement

A Thermogravimetric Analyzer (Type: Pyris 1 TGA) used the computer interface to set the parameters of the machine; the sample was placed into the platinum carrier, and then the platinum carrier was hung on the quartz hook, then the furnace was raised to cover the sample. After starting the demagnetization, the temperature was manually raised to a constant temperature and pre-baked for half an hour in a nitrogen environment to evaporate the water vapor and solvent. After confirming that the weight was stable after baking, the temperature was lowered to room temperature, and the experiment was carried out with the set heating and cooling program. After the program was completed, the weight change during the capture period was analyzed.

The measurement range of Fourier Transform infrared spectroscopy (FT-IR) can range from 2.5 μm to 25 μm (wavenumber range is 4000~600cm^−1^), and the resolution is 1 cm^−1^ to 4 cm^−1^. There are two measurement methods: the first measurement is: (1) the polymer is dissolved in tetrahydrofuran (THF) and the sulfonated polymer is dissolved in methanol (MeOH). (2) The polymer is then coated with potassium bromide (KBr) to make it dry naturally and become a membrane. (3) The thin membrane is placed into the cavity for measurement and the Fourier transform spectrum is obtained. (4) Then, qualitative and quantitative analysis is performed. Another measurement is made by (1) forming a membrane of polymer or sulfonated polymer, (2) directly placing the membrane into the cavity for measurement and obtaining the Fourier transform spectrum (3) and then performing qualitative and quantitative analysis.

## 3. Results

### 3.1. Fourier-Transform Infrared Spectroscopy Analysis [20]

**Figure 5:**
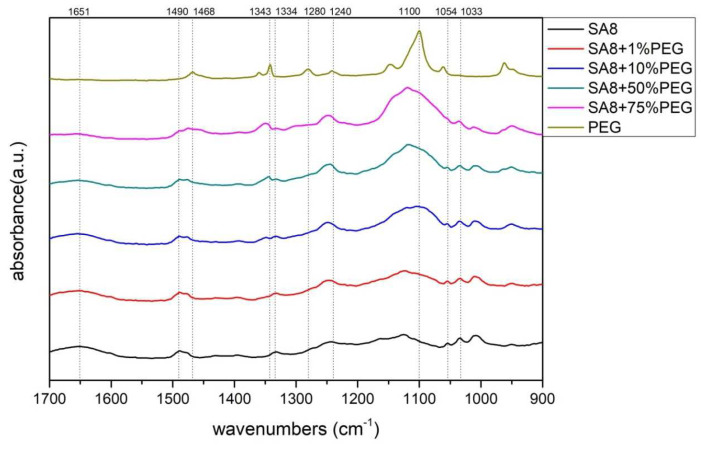
Fourier-transform infrared spectroscopy (FT-IR) spectra of SA8 blended with PEG series at 1700–900 cm^−1^.

Figure 5 shows the scanning range of SA8 blended with PEG in the range of 1700–900 cm^−1^. The O=S=O symmetrical stretching vibration peak of sulfonated SA8 at 1033 cm^−1^ proved the successful sulfonation of SA8. The symmetrical characteristic absorption peak of the ether group (ph-O-ph) of SA8 was at 1054 cm^−1^, whereas the asymmetrical characteristic absorption peaks of the ether group were at 1334 cm^−1^ and 1490 cm^−1^. The carbonyl band was near the region of 1651 cm^−1^. The stretching vibration absorption peaks of PEG hydroxyl (R-OH) were at 1100 cm^−1^, 1240 cm^−1^ and 1280 cm^−1^; the bending vibrations of C-H bond absorption peak were at 1343 cm^−1^ and 1468 cm^−1^.

**Figure 6:**
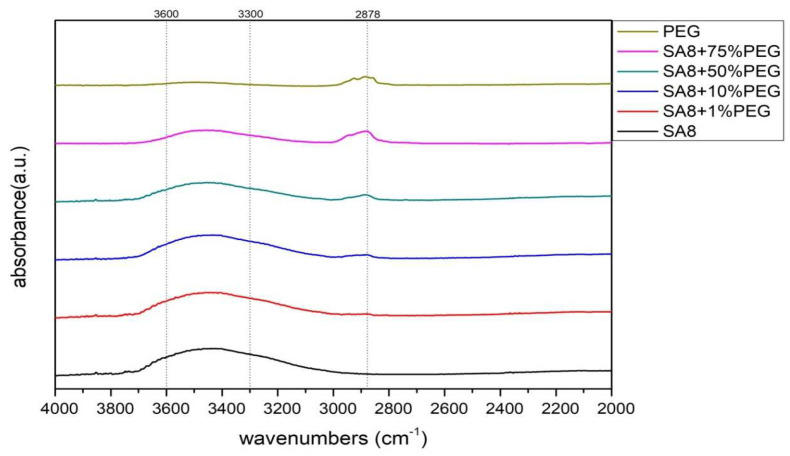
FT-IR spectra of SA8 blended with PEG series at 4000–2000 cm^−1^.

Figure 6 shows the scanning range of SA8 polymer blended with PEG enlarged in the range of 4000–2000 cm^−1^. The C-H bond stretching vibration absorption peak of PEG was at 2878 cm^−1^, and the O-H bond stretching vibration absorption peak after sulfonic acid absorption of water was in the interval 3300–3600 cm^−1^. The interaction force between the two materials is clearly observed at wavenumber positions 1100 cm^−1^ and 1334 cm^−1^. Because SA8 was subjected to the force of hydroxyl and carbon-hydrogen bonds, the wave peaks followed the blending, and gradually shifted to lower wavenumbers as the blended content increased. At the absorption peak of 2878 cm^−1^, the relationship between the blending content and the signal intensity of the aliphatic C–H bond absorption peak was also clearly observed.

**Figure 7:**
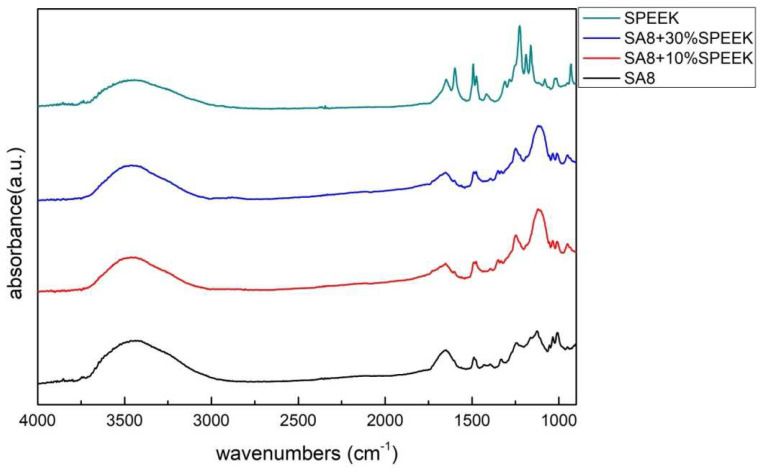
FT-IR spectra of SA8 blended with sPEEK series at 4000–900 cm^−1^.

Figure 7 shows the FT-IR spectra of the SA8 polymer blended with sPEEK at 4000–900 cm^−1^. The band at 3750–3000 cm^−1^ was the absorption peak band of both SA8 and sPEEK, and the band at this position was the stretching vibration absorption peak of the O–H bond after the sulfonic acid group had absorbed water, which proved the hydrophilicity of the polymer after sulfonation.

**Figure 8:**
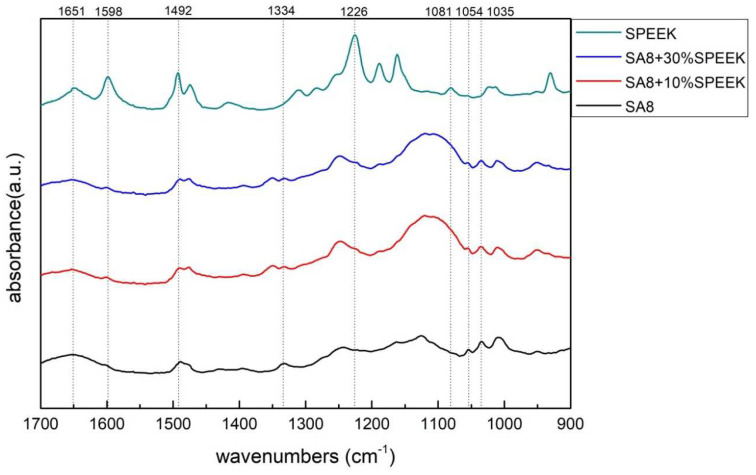
FT-IR spectra of SA8 blended with sPEEK series at 1700–900 cm^−1^.

Figure 8 shows the scanning range of SA8 polymer blended with sPEEK and enlarged to the range of 1700–900 cm^−1^. The O=S=O symmetrical stretching vibration peak of sulfonated SA8 at 1033 cm^−1^ confirmed the successful sulfonation of SA8. The symmetrical characteristic absorption peak of the ether group (ph-O-ph) of SA8 was at 1054 cm^−1^, whereas the asymmetrical characteristic absorption peaks of the ether group were at 1334 cm^−1^ and 1490 cm^−1^. The carbonyl band was near the region of 1651 cm^−1^. The sulfonated sPEEK O=S=O symmetrical stretching vibration peak at 1081 cm^−1^ confirmed the successful sulfonation of sPEEK. The PEEK aromatic C–O–C structure and sulfonated S=O stretching vibration peaks were at 1226 cm^−1^, and the C–C aromatic ring characteristic absorption peak was at 1492 cm^−1^. The C=O characteristic absorption peak was at 1598 cm^−1^, and the carbonyl band was at 1651 cm^−1^.

**Figure 9:**
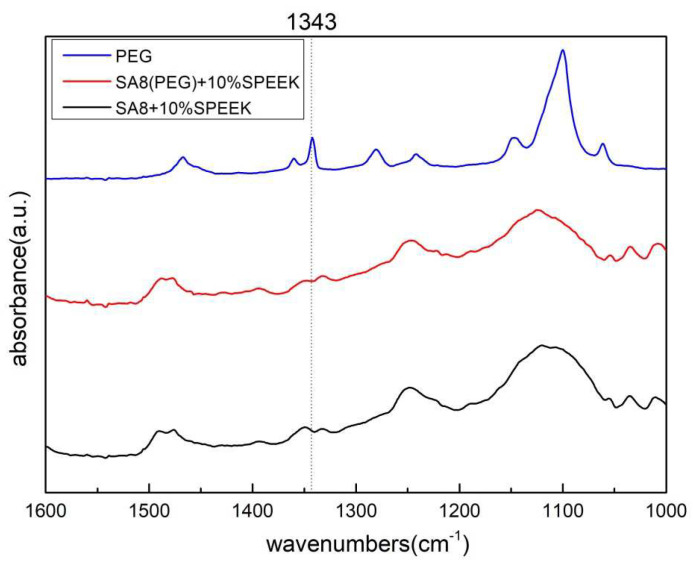
FT-IR spectra of SA8(PEG) + sPEEK blended series at 1600–1000 cm^−1^.

Figure 9 shows the scanning range of SA8 polymer blended with PEG and sPEEK when enlarged to the range of 1600–1000 cm^−1^. The flexural vibration absorption peak of the C–H bond was at 1343 cm^−1^. In this interval, the peak signal of SA8 + SPEEK composite membrane was affected by the force of the hydroxyl group of PEG and the carbon-hydrogen bond such that the wave peak shift tended to occur in the direction of lower wavenumbers.

**Figure 10:**
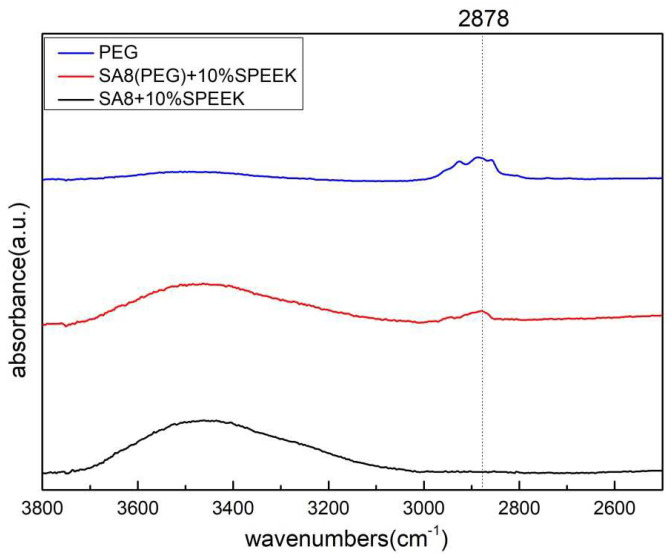
FT-IR spectra of SA8(PEG) + sPEEK blended series at 3800–2500 cm^−1^.

Figure 10 shows the scanning range of SA8 polymer blended with PEG and sPEEK when enlarged in the range of 3800–2500 cm^−1^. The absorption peak at 2878 cm^−1^ was the C–H bond stretching vibration absorption peak of PEG. The composite membrane clearly had more aliphatic C–H bond absorption peak signals of PEG.

### 3.2. Thermogravimetric Analysis of Thermal Stability

**Figure 11:**
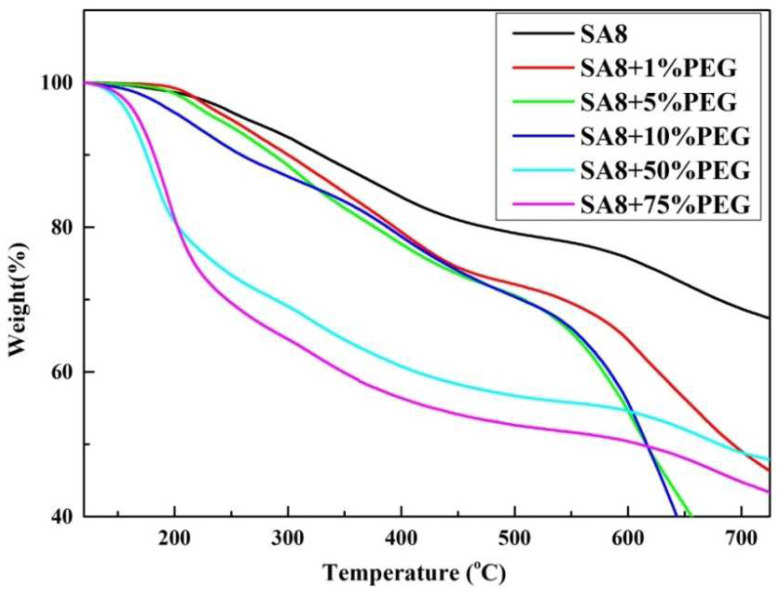
Thermogravimetric analysis (TGA) diagram of SA8 polymer blended with PEG.

As shown in Figure 11, the thermal cracking mechanism of SA8 blended with PEG mainly presents a three-stage cracking. The cracking temperature of the first stage was between 100 °C and 200 °C, and the cracking in this stage was mainly from the water molecules on the hydrophilic groups of the membrane. Because of the hydrogen bonding force between the hydrophilic group and PEG, water molecules were not easily removed by heating and remained in the film. Therefore, with the increase in the PEG content, the excessive hydrogen bonding force resulted in more water molecules, and solvents remained on the film. Moreover, excessive PEG was considerably cracked at this stage, and the thermogravimetric loss became increasingly clear with the increase in PEG blending content, and the T_d5%_ also gradually decreased. The cracking temperature of the second stage was between 200 °C and 500 °C, and the cracking at this stage was mainly from the cracking of the sulfonate group. The cracking temperature of the third stage occurred above 500 °C, which was the cracking of the main chain of the polyphenol ring structure of the SA8 polymer [21].

**Figure 12:**
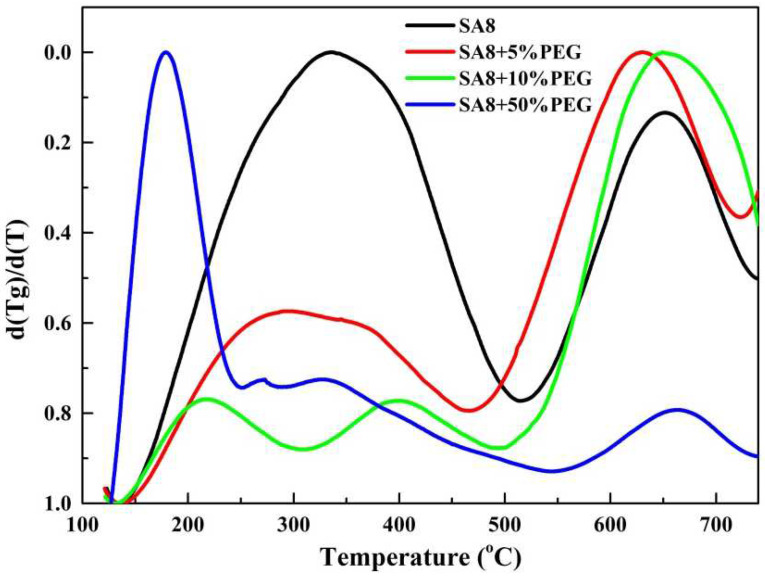
DTG diagram of SA8 polymer blended with PEG.

Figure 12 shows the derivative thermogravimetry (DTG) [22,23] diagram obtained by differentiating the thermogravimetric loss diagram of SA8 blended with PEG, in which the changes in the thermal cracking mechanism of the two materials were observed from the peaks. It was observed from the DTG diagram that SA8 had only one peak in the first stage of cracking, most of which was from the cracking of the sulfonate. However, with the addition of PEG, the wave peak gradually shifted to lower temperatures and generated a shoulder peak, which was bisected into two main peaks, suggesting that the interaction between PEG and SA8 produced other derivatives at high temperature. The thermogravimetric loss of these derivatives caused the peak to widen and gradually bisect. However, until the blending ratio was increased, a large amount of PEG cracking formed a collapsed thermogravimetric loss, and a distinct wave peak was generated at a relatively low temperature.

**Figure 13:**
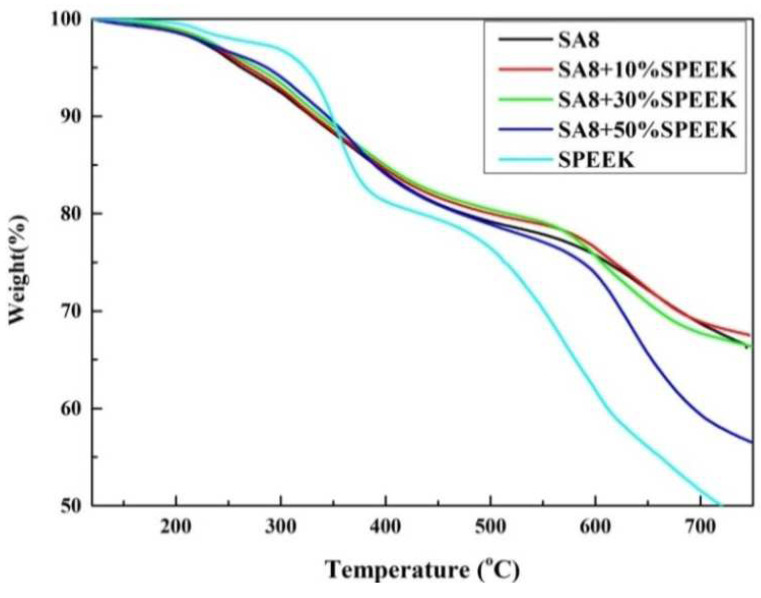
TGA diagram of SA8 polymer blended with sPEEK.

The cracking temperature of the first stage of SA8 blended with sPEEK ranged from 100 °C to 200 °C, and the cracking at this stage was mainly from the water molecules on the hydrophilic group of the membrane. Moreover, sPEEK was easier to crystallize, and the crystallinity of the material reduced the thermogravimetric loss of the membrane during combustion. Therefore, with the increase in the blending content of sPEEK, the crystallinity of the membrane increased, and the thermogravimetric loss T_d5%_ also increased gradually. Nafion between 130 and 250 °C, the loss of the –SO_3_H groups takes place [24]. With regard to SA8 blended with sPEEK, also between 200 and 500 °C, the weight loss will be around 20 wt% of the membrane, which is high for –SO_3_H groups. Moreover, the cracking temperature of the third stage occurred above 500 °C, which was not only related to polyphenol ring structure but also the sPEEK backbone (aromatic ring but without phenol), as shown in Figure 13.

**Figure 14:**
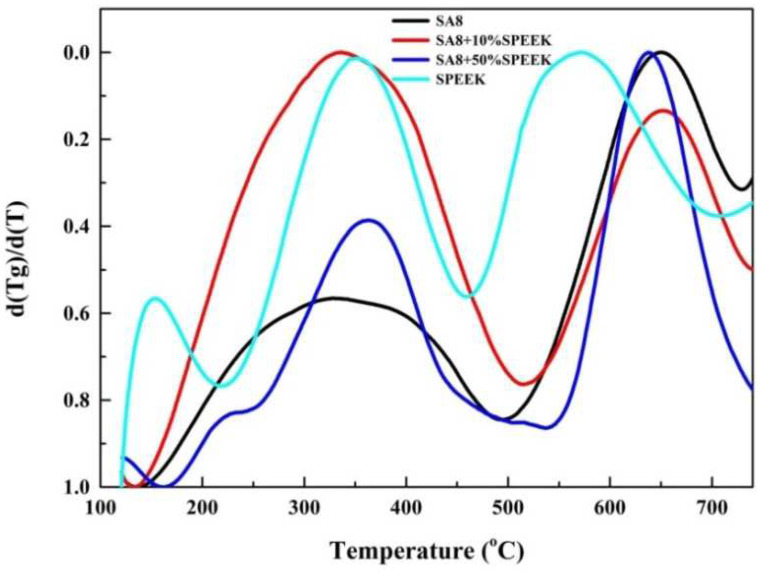
DTG diagram of SA8 polymer blended with sPEEK.

The changes in the thermal cracking mechanism of the two materials can be observed from the peaks, using the DTG diagram obtained by differentiating the thermogravimetric loss diagram of SA8 blended with sPEEK. As observed in Figure 14, SA8 had only one peak in the first stage of cracking, the majority of which was the cracking of sulfonate groups. However, with the addition of sPEEK, the wave peak gradually shifted to a higher temperature, and a shoulder peak was generated. This part indicated that the crystallinity of sPEEK started to affect the thermogravimetric loss of the film, including the changes in the cracking mechanism. Moreover, this figure shows that the cracking mechanism of sPEEK was relatively simple. The three peaks were speculated to be mainly divided into crystallization, sulfonate, and main chain cracking, and the main chain cracking temperature of sPEEK was clearly higher than that of the polyphenol ring structure of SA8.

**Figure 15:**
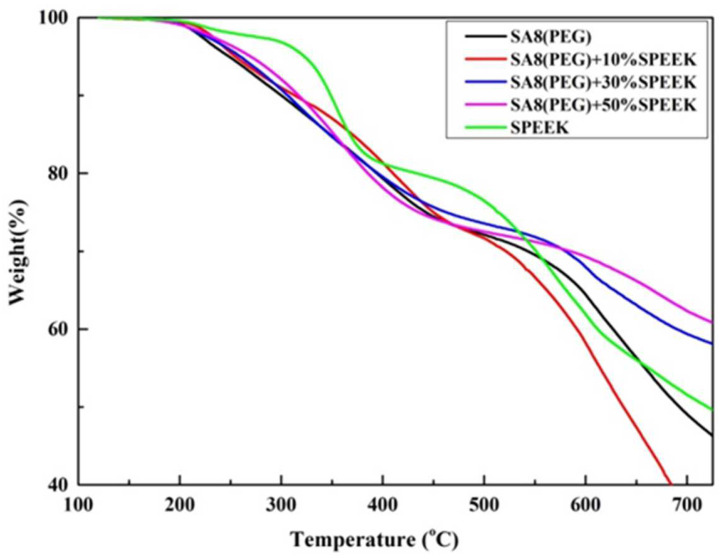
TGA diagram of SA8 polymer blended with PEG and sPEEK.

In the thermal cracking mechanism of SA8 blended with PEG and sPEEK, the cracking temperature of the first stage was between 100 °C and 200 °C, and cracking in this stage was mainly from the water molecules on the hydrophilic groups of the membrane. Moreover, sPEEK was easier to crystallize, and the crystallinity of the material reduced the thermogravimetric loss of the membrane during combustion. Therefore, with the increase in the blending content of sPEEK, the crystallization of the membrane increased, and the thermogravimetric loss T_d5%_ also gradually increased. The cracking temperature of the second stage was between 200 °C and 500 °C, and for the SA8 polymer blended with PEG and sPEEK, it was between 200 and 500 °C, therefore, the weight loss will be around 20 wt% of the membrane, which is high for –SO_3_H groups. The cracking temperature of the third stage occurred above 500°C, which was not only related to the polyphenol ring structure but also the PEG and sPEEK backbone, as shown in Figure 15.

**Figure 16:**
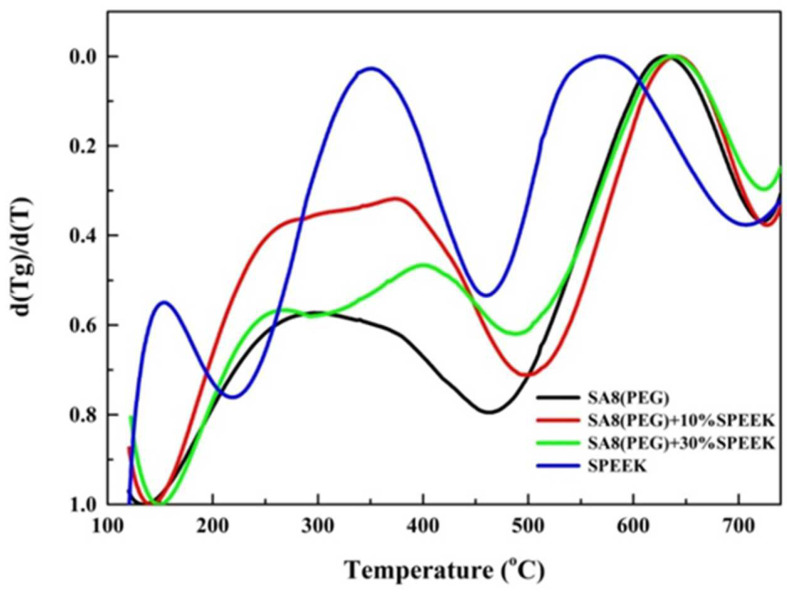
DTG diagram of sulfonated polymers [25,26,27].

The changes in the thermal cracking mechanism of the two materials were observed from the peaks in the DTG diagram obtained by differentiating the thermogravimetric loss diagram of SA8 blended with PEG and sPEEK. It was observed from Figure 16 that SA8 had a wider cracking peak in the first stage because of the additional force of PEG. In addition to the cracking of sulfonate groups, this stage also contained other derivatives generated by the interaction of the two materials. However, with the addition of sPEEK, the wave peak gradually shifted to a higher temperature, and a shoulder peak was generated. This part indicates that the crystallinity of sPEEK started to affect the thermogravimetric loss of the membrane, and that the cracking mechanism changed. After the blending ratio was increased, the segmentation of the wave peak became increasingly clear, mainly because of the cracking mechanism led by sPEEK. Finally, the main chain cracking temperature of sPEEK was significantly lower than that of the polyphenol ring structure of SA8.

### 3.3. Fuel Cell Component Performance Analysis

**Figure 17:**
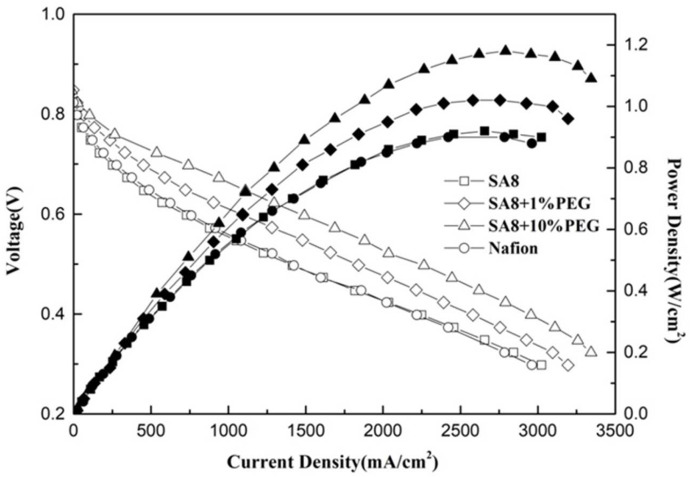
Fuel cell component performance diagram of SA8 blended with PEG.

**Figure 18:**
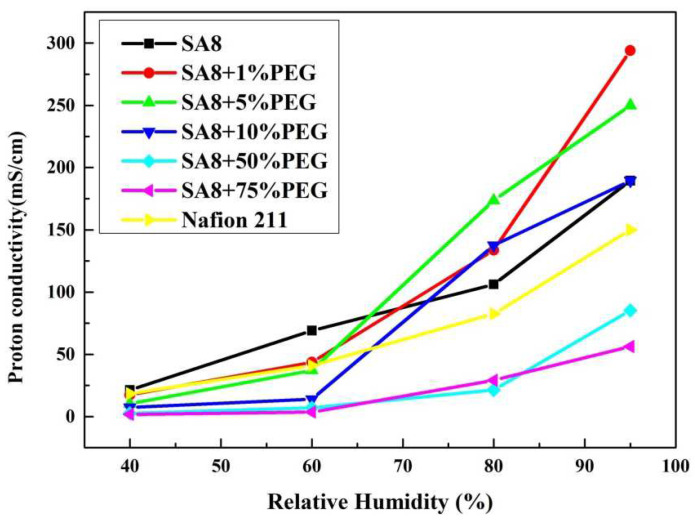
The proton conductivity diagram of SA8 blended with PEG.

**Figure 19:**
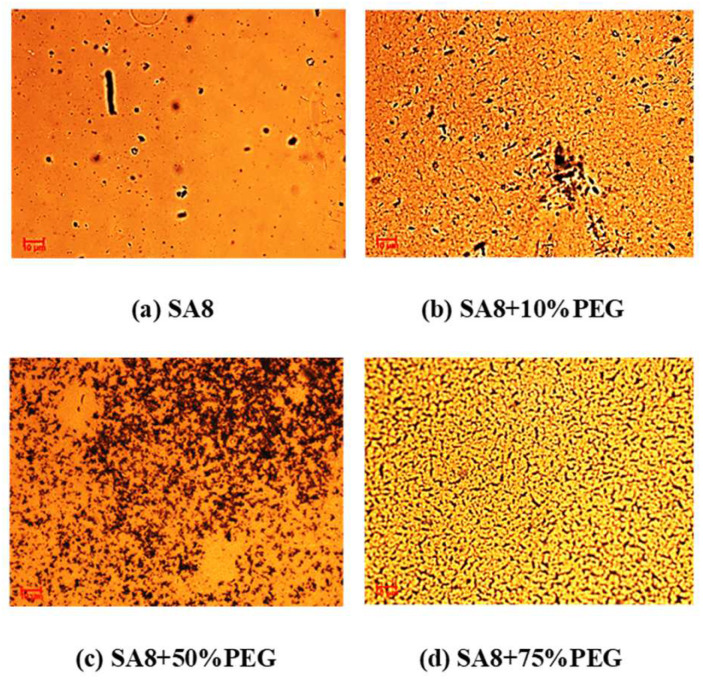
The morphology diagram of SA8 blended with PEG.

Figure 17 shows the performance diagram of the fuel cell component [28,29] after SA8 was blended with PEG, and the measurement conditions and performance of the components are summarized in Table 5. After SA8 was blended with PEG, the performance of its fuel cell components had a chemical stability. From this tendency, it was observed that the hydrogen bonding force between PEG and sulfonate had a significant impact on the membrane characteristics. The addition of an appropriate amount of PEG improved the dimensional stability, which in turn, optimized the component performance of the membrane. Although SA8 with 1% PEG had the best proton conductivity, the component performance was lower than that of SA8 with 10% PEG, as shown in Figure 18. The physical crosslinking of 10% PEG probably resulted in a better molecular arrangement of the membrane. The arrangement also altered the phase separation pattern and transport mechanism of the membrane, thereby improving the component performance of the membrane. Moreover, as PEG has a plasticizing function, the introduction of PEG increased the flexibility of the film, which resulted in better adhesion of the membrane electrode assembly of the component, such that a better component performance was achieved. We observed the mixed morphology of the two materials through a polarizing microscope. When PEG is added to 50%, it becomes a phase separation type with obvious direct stratification, as shown in Figure 19.

**Figure 20:**
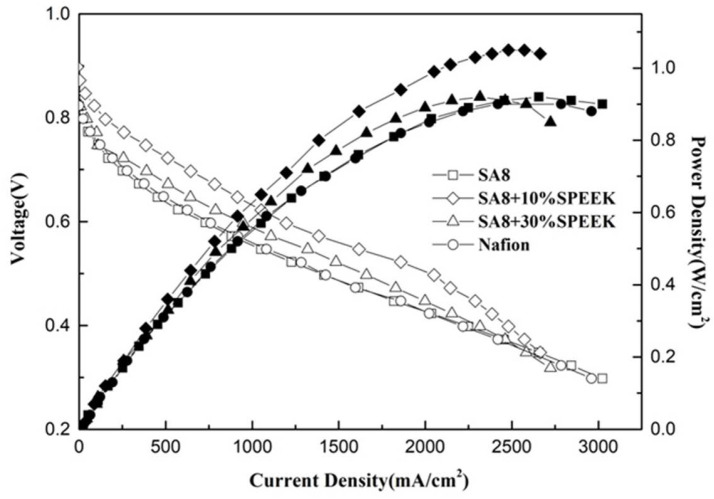
Fuel cell component performance diagram of SA8 blended with sPEEK.

**Figure 21:**
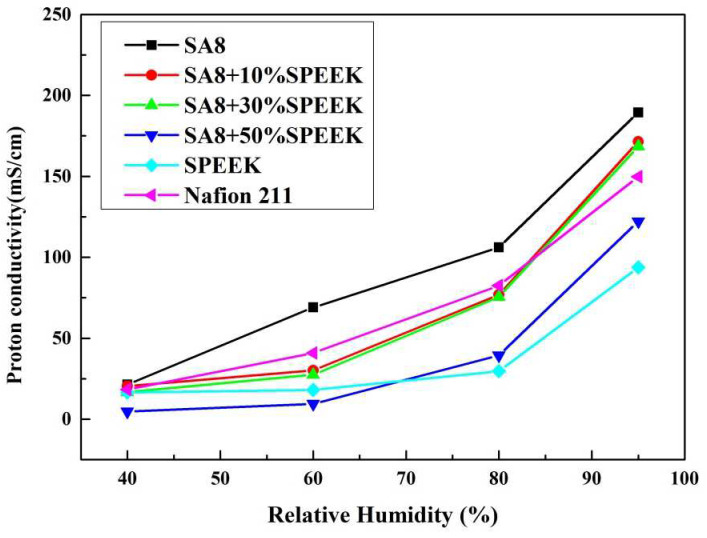
The proton conductivity diagram of SA8 blended with sPEEK.

**Figure 22:**
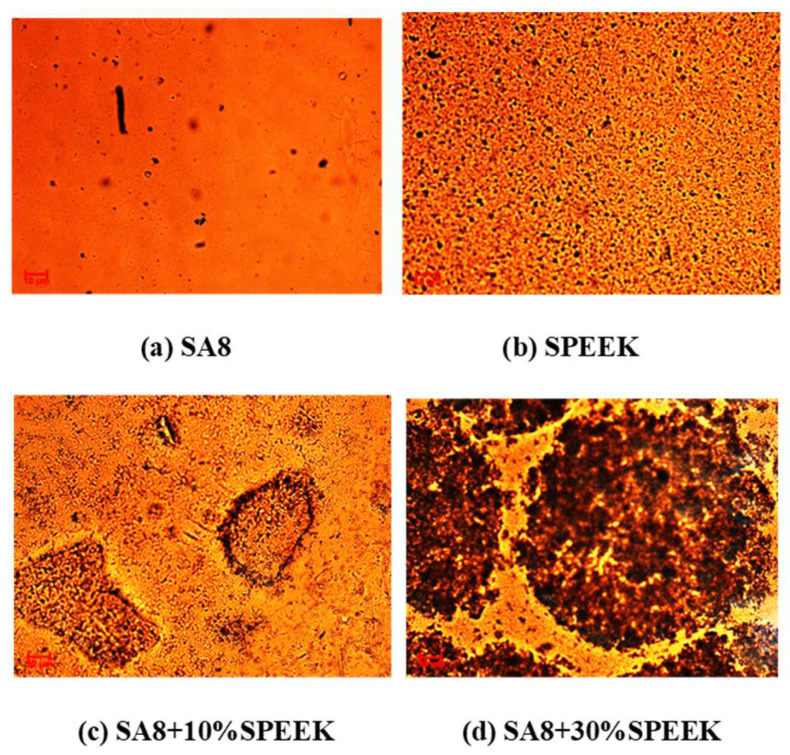
The morphology diagram of SA8 blended with sPEEK.

Figure 20 shows the performance diagram of the fuel cell components after SA8 was blended with sPEEK, and the measurement results of the components are summarized in Table 6. After SA8 was blended with 10% sPEEK, the performance of the fuel cell component was optimized. The proton conductivity diagram of SA8 blended with sPEEK is shown in Figure 21. From this phenomenon, it was observed that the excellent dimensional stability of sPEEK had a significant impact on the characteristics of the membrane; after blending with sPEEK, the phase separation pattern of the membrane was changed because of the crystallinity of the material itself. The morphology diagram of SA8 blended with sPEEK is shown in Figure 22. However, as the sPEEK content increased, the component performance decreased. As the crystalline clusters inside the film became larger, blocking the proton transmission channels, and the number of sulfonate groups in the membrane decreased, the water absorption capacity became poorer, and the carrier for transporting protons decreased, which reduced the component performance of the membrane.

**Figure 23:**
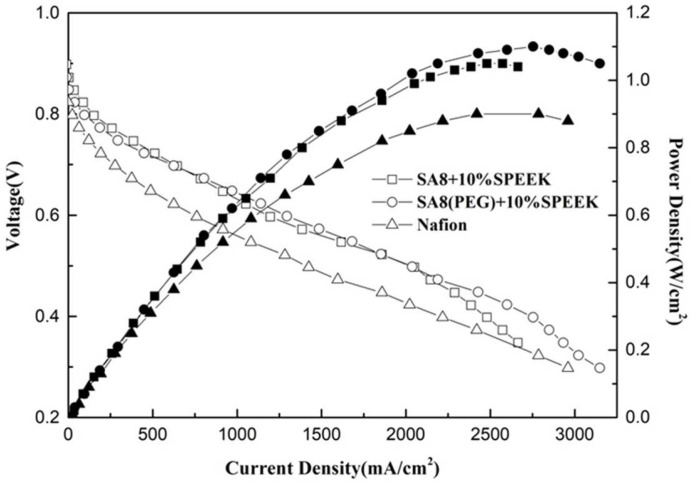
Fuel cell component performance diagram of SA8 blended with PEG and 10% sPEEK.

**Figure 24:**
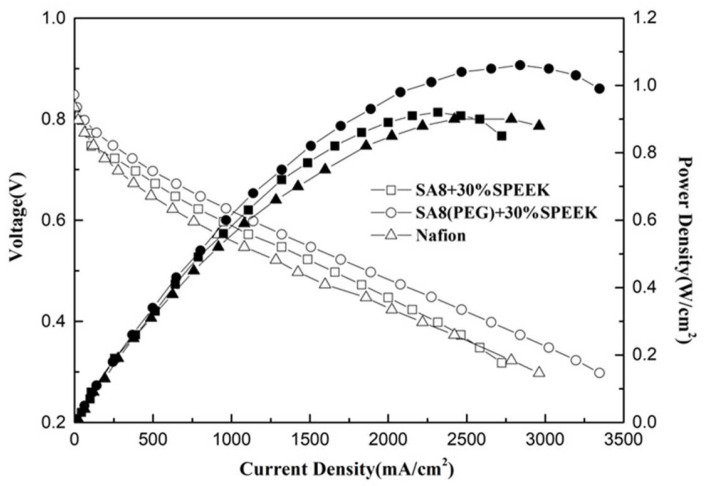
Fuel cell component performance diagram of SA8 blended with PEG and 30% sPEEK.

**Figure 25:**
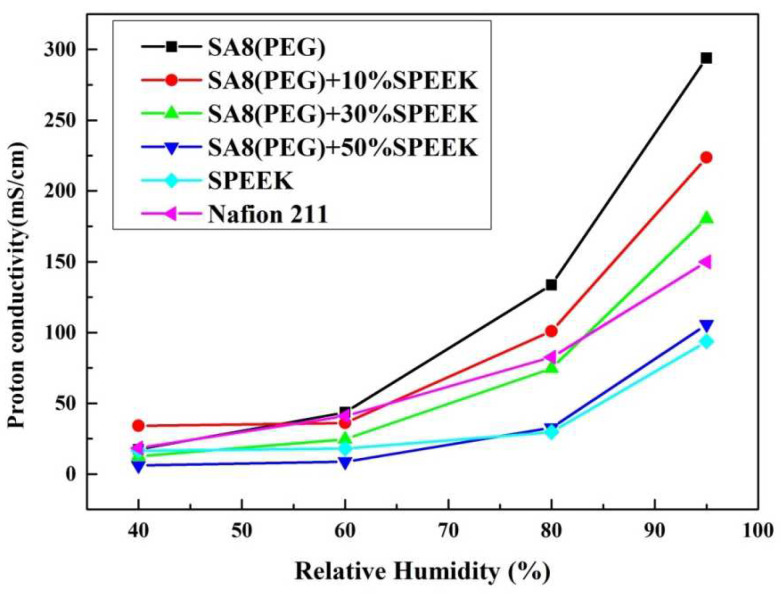
The proton conductivity diagram of SA8 blended with PEG and sPEEK.

**Figure 26:**
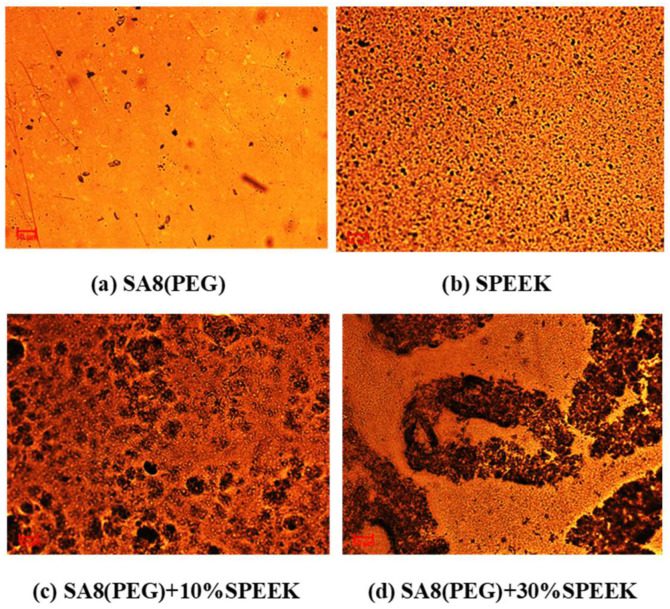
The morphology diagram of SA8 blended with PEG and sPEEK.

Figure 23 shows the performance diagram of the fuel cell component of SA8 blended with PEG and 10% sPEEK, and Figure 24 shows the performance diagram of the fuel cell component of SA8 polymer blended with PEG and 30% sPEEK. The proton conductivity diagram of SA8 blended with PEG and sPEEK is shown in Figure 25. The measurement results of blending 10% sPEEK and 30% sPEEK are summarized in Table 7. The excellent dimensional stability of sPEEK had a considerable impact on the membrane characteristics, and the phase separation pattern of the membrane was altered because of the crystallinity of the material. However, as the sPEEK content increased, the component performance also decreased. Because the crystalline clusters inside the membrane became larger, blocking the proton transmission channels, the number of sulfonate groups in the membrane decreased, and the water absorption capacity became poorer, such that the carrier for transporting protons decreased, which reduced the component performance of the membrane, as shown in Table 8. We formulated Fenton’s reagent to test the chemical stability of the membranes. The chemical stability of SA8 blended with PEG and sPEEK is shown in Table 9. After adding PEG to the SA8 + sPEEK composite membrane, the component performance demonstrated the chemical stability. This tendency validated that the addition of PEG had an absolute effect on the interaction between SA8 and sPEEK. The results showed that physical cross-linking improved the molecular arrangement of the membrane, which altered its phase separation pattern and transport mechanism, thus, improving the component performance. The morphology diagram of SA8 blended with PEG and sPEEK is shown in Figure 26.

## 4. Conclusions

The SA8 polymer blended with PEG composite membrane was observed under FT-IR structural identification. The characteristic absorption peaks of the sulfonated polymer SA8 and PEG were shifted, and the shift intensity varied with different blending ratios [30]. The comparison of the peak spectra of the references confirmed that the hydrogen bonding force between the two materials was closely related to the C-H bond of PEG [31] and the ether group of SA8. The device performance was significantly improved by blending SA8 polymer with PEG and was higher than that of Nafion 211′s 0.92 W/cm^2^. The best performance of 1.18 W/cm^2^ was obtained for SA8 blended with 10% PEG. Material identification of the SA8 polymer blended with sPEEK composite membrane was performed using FT-IR, confirming the analytical results by comparing with the absorption peak spectrum of the reference. In terms of film morphology, the film contained small speckled crystals because of the easy crystallization (crystallinity) of sPEEK. From the observation results of the polarized light microscope, with the increase in sPEEK content, the crystalline clusters of the film were also increasingly larger. The crystalline nature resulted in significant changes in some properties. The composite membrane of SA8 polymer blended with 10% sPEEK improved the component performance. The appropriate proportion of sPEEK blending apparently seems to have a certain effect on the phase separation morphology of the film. Although the crystalline nature of the membrane hindered the transmission channel of the film and considerably reduced the proton conductivity, the regular crystalline distribution of the film also resulted in different phase separation morphologies, which enabled the component performance of the composite membrane to be considerably optimized, with the highest performance of up to 1.05 W/cm^2^, higher than that of Nafion 211’s 0.92 W/cm^2^. Subsequently, we investigated the material identification of SA8 blended with PEG and composite membranes blended with 10% sPEEK and 30% sPEEK using FT-IR and confirmed the analytical results by comparing the absorption peak spectra of the references. In terms of film morphology, the results of polarized light microscope observation showed that with the increase in sPEEK content, the crystalline clusters of the membrane became increasingly larger. The crystallinity of sPEEK resulted in significant changes in some properties of the membrane. In terms of water absorption capacity and dimensional stability, as the blending ratio of sPEEK with a low degree of sulfonation increased, the water absorption capacity of the composite membrane decreased, which caused a decrease in the rate of dimensional change. In terms of thermal stability, due to the crystallinity of the sPEEK material itself, the thermal cracking temperature of the film increased with the increase in the blending ratio, and the crystalline clusters became larger, which increased the thermal cracking temperature. In the measurement of the component performance of SA8 blended with PEG and composite membranes blended with 10% sPEEK and 30% sPEEK, it was observed that the SA8 + sPEEK composite membrane blended with PEG improved the component performance. The reason was that the appropriate blending ratio of PEG changed the molecular arrangement of the membrane such that the component performance decreased with the increase in sPEEK content, even with the transmission channel of the membrane hindered by the crystallinity of sPEEK. The introduction of PEG improved the component performance of the composite membrane, and its performance was considerably improved.

## 5. Patents

Patents resulting from the work reported in this manuscript: US8,987,407B2; US9,748,594B2; US9,644,069B2; US9,209,472B2; US9,018,336B2.

## Figures and Tables

**Table 1:** Solid contents of SA8 and PEG.

Sulfonated Polymer	Solid Content of SA8	Solid Content of PEG
**100%SA8**	1.000 g	0.000 g
**99%SA8 + 1%PEG**	0.990 g	0.004 g
**95%SA8 + 5%PEG**	0.950 g	0.020 g
**90%SA8 + 10%PEG**	0.900 g	0.040 g
**50%SA8 + 50%PEG**	0.500 g	0.200 g
**25%SA8 + 75%PEG**	0.250 g	0.300 g

$\mathrm{Solid}\;\mathrm{content}\;\mathrm{of}\;\mathrm{SA}8=\mathrm{Weight}\;\mathrm{of}\;\mathrm{film}-\mathrm{forming}\;\mathrm{solution}\;\left(8\;g\right)\times \mathrm{blending}\;\mathrm{ratio}\times 12.5\%;$ $\mathrm{Solid}\;\mathrm{content}\;\mathrm{of}\;\mathrm{PEG}=\mathrm{Weight}\;\mathrm{of}\;\mathrm{film}-\mathrm{forming}\;\mathrm{solution}\;\left(8\;g\right)\times \mathrm{blending}\;\mathrm{ratio}\times 5\%.$

**Table 2:** Solid contents of SA8 and sPEEK.

Sulfonated Polymer	Solid Content of SA8	Solid Content of sPEEK
**100%SA8**	1.00 g	0.00 g
**90%SA8 + 10%SPEEK**	0.09 g	0.04 g
**70%SA8 + 30% SPEEK**	0.70 g	0.12 g
**50%SA8 + 50% SPEEK**	0.50 g	0.20 g
**100%SPEEK**	0.00 g	0.40 g

$\mathrm{Solid}\;\mathrm{content}\;\mathrm{of}\;\mathrm{SA}8=\mathrm{Weight}\;\mathrm{of}\;\mathrm{film}-\mathrm{forming}\;\mathrm{solution}\;\left(8\;g\right)\times \mathrm{blending}\;\mathrm{ratio}\times 12.5\%;$ $\mathrm{Solid}\;\mathrm{content}\;\mathrm{of}\;\mathrm{PEG}=\mathrm{Weight}\;\mathrm{of}\;\mathrm{film}-\mathrm{forming}\;\mathrm{solution}\;\left(8\;g\right)\times \mathrm{blending}\;\mathrm{ratio}\times 5\%.$

**Table 3:** Solid contents of SA8 and sPEEK.

Sulfonated Polymer	Solid Content of SA8	Solid Content of PEG
**SA8(PEG)**	4.95 g	0.02 g

**Table 4:** Solid contents of SA8(PEG) and sPEEK.

Sulfonated Polymer	Weight of SA8(PEG) Solution	Weight of sPEEK Solution
**100%SA8(PEG)**	8.0 g	0.0 g
**90%SA8(PEG) + 10%SPEEK**	7.2 g	0.8 g
**70%SA8(PEG) + 30%SPEEK**	5.6 g	2.4 g
**50%SA8(PEG) + 50%SPEEK**	4.0 g	4.0 g
**100%SPEEK**	0.0 g	8.0 g

**Table 5:** Component measurement conditions and performance of SA8 blended with PEG.

Sulfonated Polymer	Anode Loading (mg/cm^2^)	Cathode Loading (mg/cm^2^)	Open Circuit Voltage (V)	Power Density (W/cm^2^)
**SA8**	0.2	0.4	0.958	0.92
**SA8 + 1%PEG**	0.2	0.4	0.962	1.02
**SA8 + 10%PEG**	0.2	0.4	0.885	1.18
**Nafion 211**	0.2	0.4	0.955	0.90

**Table 6:** Component measurement conditions and performance of SA8 blended with sPEEK.

Sulfonated Polymer	Anode Loading (mg/cm^2^)	Cathode Loading (mg/cm^2^)	Open Circuit Voltage (V)	Power Density (W/cm^2^)
**SA8**	0.2	0.4	0.958	0.92
**SA8 + 10%SPEEK**	0.2	0.4	0.963	1.05
**SA8 + 30%SPEEK**	0.2	0.4	0.960	0.92
**Nafion 211**	0.2	0.4	0.955	0.90

**Table 7:** Component measurement conditions and performance of SA8 blended with PEG and 10% sPEEK and 30% sPEEK.

Sulfonated Polymer	Anode Loading (mg/cm^2^)	Cathode Loading (mg/cm^2^)	Open Circuit Voltage (V)	Power Density (W/cm^2^)
**SA8 + 10%SPEEK**	0.2	0.4	0.963	1.04
**SA8(PEG) + 10%SPEEK**	0.2	0.4	0.947	1.10
**SA8 + 30%SPEEK**	0.2	0.4	0.960	0.92
**SA8(PEG) + 30%SPEEK**	0.2	0.4	0.965	1.06
**Nafion 211**	0.2	0.4	0.955	0.90

**Table 8:** The physicochemical parameter characteristics of SA8 blended with PEG and sPEEK.

Sulfonated Polymer	Water Uptake (%)	Dimensional Swelling (L%)	Dimensional Swelling (W%)	Hydration Number (λ)
**SA8(PEG)**	82	12.50	12.82	15.3
**SA8(PEG) + 10%SPEEK**	63.9	11.25	10.00	12.1
**SA8(PEG) + 30%SPEEK**	73.7	8.75	10.00	14.8
**SA8(PEG) + 50%SPEEK**	54	8.75	7.14	10.6
**SPEEK**	17.9	5.00	5.00	8.6
**Nafion 211**	33.3	7.50	7.50	20.3

**Table 9:** The chemical stability of SA8 blended with PEG and sPEEK.

Sulfonated Polymer	Hydrolytic Stability (%)	Oxidative Stability (%)
**SA8**	98.4%	87.4%
**SA8(PEG)**	99.1%	92.7%
**SA8(PEG) + 10%SPEEK**	99.3%	95.5%
**SA8(PEG) + 30%SPEEK**	98.7%	94.5%
**SA8(PEG) + 50%SPEEK**	98.5%	96.8%
**SPEEK**	100.0%	57.3%

Hydrolytic stability was measuring at 100 °C for 24 h. Oxidative stability was measuring at 80 °C for 1 h.

## Data Availability

Not applicable.
